# Sensitivity of the Boston criteria version 2.0 in Dutch-type hereditary cerebral amyloid angiopathy

**DOI:** 10.1177/17474930241239801

**Published:** 2024-03-21

**Authors:** RGJ van der Zwet, EA Koemans, S Voigt, R van Dort, I Rasing, K Kaushik, TW van Harten, MR Schipper, GM Terwindt, MJP van Osch, MAA van Walderveen, ES van Etten, MJH Wermer

**Affiliations:** 1 Department of Neurology, Leiden University Medical Center, Leiden, The Netherlands; 2 Department of Radiology, Leiden University Medical Center, Leiden, The Netherlands; 3 Department of Neurology, University Medical Center Groningen, Groningen, The Netherlands

**Keywords:** Intracerebral hemorrhage, cerebral amyloid angiopathy, small vessel disease

## Abstract

**Background and aim:**

The revised Boston criteria v2.0 for cerebral amyloid angiopathy (CAA) add two radiological markers to the existing criteria: severe visible perivascular spaces in the centrum semiovale and white matter hyperintensities (WMHs) in a multispot pattern. This study aims to determine the sensitivity of the updated criteria in mutation carriers with Dutch-type hereditary CAA (D-CAA) in an early and later disease stage.

**Methods:**

In this cross-sectional study, we included presymptomatic and symptomatic D-CAA mutation carriers from our prospective natural history study (AURORA) at the Leiden University Medical Center between 2018 and 2021. 3-Tesla scans were assessed for CAA-related magnetic resonance imaging (MRI) markers. We compared the sensitivity of the Boston criteria v2.0 to the previously used modified Boston criteria v1.5.

**Results:**

We included 64 D-CAA mutation carriers (mean age 49 years, 55% women, 55% presymptomatic). At least one white matter (WM) feature was seen in 55/64 mutation carriers (86%: 74% presymptomatic, 100% symptomatic). Fifteen (23%) mutation carriers, all presymptomatic, showed only WM features and no hemorrhagic markers. The sensitivity for probable CAA was similar between the new and the previous criteria: 11/35 (31%) in presymptomatic mutation carriers and 29/29 (100%) in symptomatic mutation carriers. The sensitivity for possible CAA in presymptomatic mutation carriers increased from 0/35 (0%) to 15/35 (43%) with the new criteria.

**Conclusion:**

The Boston criteria v2.0 increase the sensitivity for detecting possible CAA in presymptomatic D-CAA mutation carriers and, therefore, improve the detection of the early phase of CAA.

## Introduction

Sporadic cerebral amyloid angiopathy (CAA) is a common cerebral small vessel disease for which a neuropathological examination is necessary to establish a definite diagnosis. The modified Boston criteria (v1.5) have been widely used to diagnose possible and probable CAA during life based on clinical and radiological criteria.^1^ The criteria mainly relied on hemorrhagic magnetic resonance imaging (MRI) markers and were developed in a population consisting primarily of patients with intracerebral hemorrhage (ICH). Over the years, however, evidence accumulated that non-hemorrhagic markers may also be important. Also, the clinical spectrum of CAA has turned out to be broad, ranging from ICH to cognitive decline and transient focal neurological episodes (TFNEs).^2^ The Boston criteria were recently updated (v2.0) to address these new insights by (1) adding non-hemorrhagic markers, namely, severe visible perivascular spaces in the centrum semiovale and white matter hyperintensities (WMHs) in a multispot pattern to the criteria and (2) performing a validation in cohorts that also included non-ICH CAA patients.^3^ Nevertheless, this validation was limited due to the selection of patients with severe CAA pathology. As a result, the performance of the criteria in patients with early or mild signs of CAA is unknown.

Dutch-type hereditary CAA (D-CAA) is an autosomal dominant hereditary form of CAA with similar symptoms, MRI findings, and pathology, albeit with an earlier and more rapidly progressing disease course.^4,5^ Due to these similarities, D-CAA can function as a model for studying CAA in a population with a limited influence of comorbidities. Unlike the neuropathologic confirmation needed in an sCAA cohort, genetic testing confirms a definite D-CAA diagnosis during life, creating the opportunity to investigate the early, presymptomatic disease phase.

## Aim

We aimed to investigate whether the sensitivity of the Boston criteria v2.0 is improved compared with v1.5 in both presymptomatic and symptomatic D-CAA mutation carriers.

## Methods

### Patient selection

We included mutation carriers with D-CAA from our prospective natural history study (AURORA) consecutively at the Leiden University Medical Center between 2018 and 2021. D-CAA was diagnosed by genetic testing. Mutation carriers with a history of ⩾1 symptomatic ICH were classified as symptomatic. We retrieved information on medical history and vascular risk factors from medical records. Participants were excluded when no MRI was available. The AURORA study was approved by the Medical Ethics Committee Leiden Den Haag Delft (approval no. NL62670.058.17/ P17.235), and written informed consent was obtained from all participants.

### MR: image acquisition

All patients were scanned using a whole-body magnetic resonance system with a 3T field strength (Philips Medical Systems, Best, The Netherlands). We obtained T2-weighted images (echo time (TE) 80 ms, repetition time (TR) 4744 ms, flip 90°, 48 slices, field of view (FOV) 220 × 176 × 144 mm, slice thickness 3.00 mm, voxel size 0.5 × 0.6 × 3.0 mm, and scan duration ∼2 min), fluid-attenuated inversion recovery (FLAIR; TE 280 ms, TR 4800 ms, inversion time (TI): 1650 ms, 321 slices, FOV 250 × 250 × 180 mm, voxel size 1.0 × 1.0 × 0.6 mm, and scan duration ~5 min), and susceptibility-weighted imaging (SWI; TE 31 ms, TR 7.2 ms, flip 17°, 130 slices, FOV 230 × 190 × 130 mm, voxel size 0.6 × 0.6 × 1.0 mm, and scan duration ∼4 min).

### Radiological assessment

Images were scored for the presence of CAA-related disease markers according to the *Standards for Reporting Vascular Changes on Neuroimaging Criteria* (STRIVE-2).^6^ The following markers were scored: cerebral hemorrhages (cerebral microbleeds (CMBs) and macrobleeds), cortical superficial siderosis (cSS), severe visible perivascular spaces in the centrum semiovale, and WMH in a multispot pattern.

Cerebral macrobleeds were defined as hypointense large (diameter > 10 mm) lesions with either an irregular shape or a cystic cavity on SWI.^7^ CMBs were defined as small (2–10 mm in diameter) areas of signal void with associated blooming on SWI.^6^ cSS was defined as curvilinear hypointensities following the cortical surface, distinct from the vessels on SWI.^6^ Acute convexity subarachnoid hemorrhage (cSAH) was defined as a linear hypointensity in the subarachnoid space affecting one or more cortical sulci on SWI sequences with corresponding hyperintensity in the subarachnoid space on T1-weighted or FLAIR images.^7^

Severe visible perivascular spaces in the centrum semiovale were defined as >20 visible linear, round, or ovoid spaces in the centrum semiovale, with signal intensity similar to cerebrospinal fluid (CSF) on T2-weighted imaging.^6^ WMHs in a multispot pattern were defined as the presence of >10 small circles or spots of WMH on FLAIR.^3^ T2-weighted imaging was used when no FLAIR MRI was available (n = 19).

Three independent observers (R.G.J.v.d.Z., R.v.D., and E.A.K.) assessed the available MRI scans for CAA-related small vessel disease markers according to the Boston v2.0 criteria.^3^ One observer (R.G.J.v.d.Z., 2 years of experience in the field) scored the WMH multispot pattern, and another observer (R.v.D., 2 years of experience in the field) performed a 20% cross-check. Discrepancies were discussed with a neuroradiologist (M.A.A.v.W., >15 years of experience in the field). The other markers were scored by another observer (E.A.K., 5 years of experience in the field).

Based on the presence of these markers, all subjects were subdivided into categories “possible CAA,” “probable CAA,” and “no CAA,” according to the Boston criteria v1.5 and v2.0.

### Statistics

The frequency of CAA markers was reported as proportions. The sensitivity was calculated by dividing the number of mutation carriers correctly diagnosed with CAA according to the criteria by the total number of mutation carriers. Statistical analyses were performed with the Statistical Package of Social Sciences (SPSS), Version 26. The study was conducted in accordance with the Standards for Reporting of Diagnostic Accuracy Studies 2015 (STARD) guidelines.^8^

## Results

**Figure 1.**
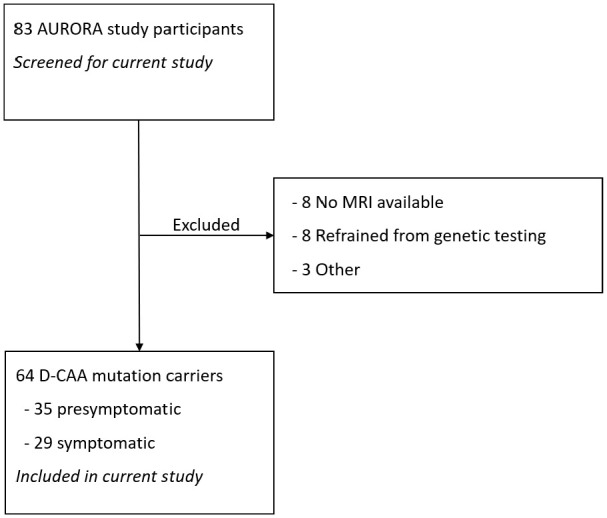
Flowchart of patient selection.

We included 64 D-CAA mutation carriers (mean age 49 years, 55% women, 35 (55%) presymptomatic, Table 1 and Figure 1). Fourteen (22%) had hypertension, and 41 (64%) had a history of smoking.

**Table 1.** Age, sex, and frequency of MRI markers.

Characteristic	All (n = 64)	Presymptomatic (n = 35)	Symptomatic (n = 29)
Age, mean (SD), y	49.1 (12.2)	42.1 (11.5)	57.6 (6.2)
Female sex (%)	35 (55%)	22 (63%)	13 (49%)
Presence of hemorrhagic markers (%)	40 (63%)	11 (31%)	29 (100%)
Lobar cerebral microbleeds	40 (63%)	11 (31%)	29 (100%)
Lobar macrobleeds	33 (50%)	4 (11%)	29 (100%)
Cortical superficial siderosis	20 (31%)	2 (6%)	18 (62%)
Convexity subarachnoid hemorrhage	0 (0%)	0 (0%)	0 (0%)
Presence of white matter features (%)	55 (86%)	26 (74%)	29 (100%)
Only multispot pattern	4 (6%)	2 (6%)	2 (7%)
Only severe visible perivascular spaces in the centrum semiovale	10 (16%)	10 (29%)	0 (0%)
Both	41 (64%)	14 (40%)	27 (93%)

Twenty-six (74%) of the 35 presymptomatic mutation carriers had at least one white matter feature (Table 1). Eleven (31%) presymptomatic mutation carriers had hemorrhagic lesions on MRI (all multiple, all with at least one white matter feature). Four (11%) presymptomatic mutation carriers had asymptomatic lobar macrobleeds incidentally found on MRI. All symptomatic patients had multiple hemorrhagic lesions on MRI and at least one white matter feature. The youngest mutation carrier with WMH in a multispot pattern was 30 years, and the youngest mutation carrier with severe visible perivascular spaces in the centrum semiovale was 29 years old.

### Sensitivity of the Boston criteria v2.0

**Figure 2.**
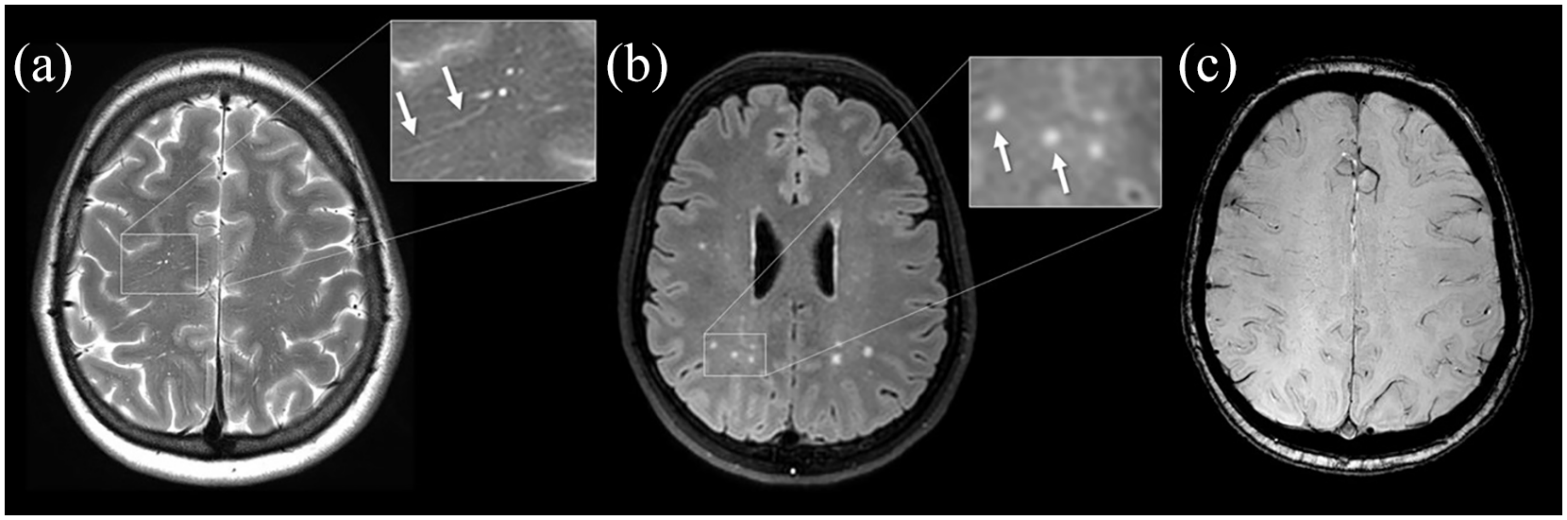
Patient with possible CAA according to the Boston criteria v2.0. Presymptomatic woman with D-CAA with severe (>20 in one hemisphere) visible perivascular spaces in the centrum semiovale on T2-weighted imaging (a, arrows), WMHs in a multispot pattern on FLAIR (b, arrows), and no hemorrhagic MRI markers on SWI (c). This patient fulfills the Boston criteria v2.0 but not the modified Boston criteria v1.5 for possible CAA.

The sensitivity for a diagnosis of probable CAA was similar for both the Boston criteria v1.5 and v2.0: 11/35 (31%) in presymptomatic mutation carriers and 29/29 (100%) in symptomatic mutation carriers (Table 2). The Boston criteria v2.0 increased the sensitivity for possible CAA from 0/35 (0%) to 15/35 (43%) in presymptomatic mutation carriers compared to v1.5. An example of one patient who was newly classified as “possible CAA” can be seen in Figure 2. All mutation carriers with possible CAA according to only the Boston criteria v2.0 had at least one white matter feature but no hemorrhagic features. No new mutation carriers met the criteria for probable CAA using the Boston criteria v2.0 compared to v1.5 because all mutation carriers with hemorrhagic markers already had at least two hemorrhagic markers on MRI.

**Table 2.** The sensitivity of modified v1.5 and new Boston v2.0 criteria.

	Probable CAA (vs non-probable CAA)		Possible CAA (vs no CAA)		Any CAA (probable + possible, vs no CAA)	
	Boston criteria v1.5	Boston criteria v2.0	Boston criteria v1.5	Boston criteria v2.0	Boston criteria v1.5	Boston criteria v2.0
Presymptomatic (n = 35)	11/35 (31%)	11/35 (31%)	0/35 (0%)	15/35 (43%)	11/35 (31%)	26/35 (74%)
Symptomatic (n = 29)	29/29 (100%)	29/29 (100%)	0/0 (-)	0/0 (-)	29/29 (100%)	29/29 (100%)
Total (n = 64)	40/64 (63%)	40/64 (63%)	0/35 (0%)	15/35 (43%)	40/64 (63%)	55/64 (86%)

## Discussion

We found that the Boston criteria v2.0 increase the sensitivity for detecting possible CAA in presymptomatic mutation carriers with D-CAA. There was no additional value in patients with advanced D-CAA who had hemorrhagic markers on MRI because all of these patients already had multiple hemorrhages. Our results confirm the expectation that the Boston criteria v2.0 are more suitable for detecting the early stage of CAA.

One other study validated the Boston criteria v2.0.^9^ In this study of 122 patients with lobar ICH, the proportion of patients diagnosed with CAA increased from 30% to 39% when using the Boston criteria v2.0. This study, however, did not investigate patients in the early phases of CAA or patients with a definite diagnosis of CAA. Another study showed that patients classified as probable CAA according to the Boston criteria v2.0 alone have a greatly reduced risk of recurrent ICH when compared to patients classified using the Boston criteria v1.5, comparable to patients with ICH and no signs of CAA.^10^ These findings suggest that patients identified only by the new criteria have less severely advanced CAA and may even reflect the reduced specificity of the new criteria in patients who present with ICH.

Our study has limitations. We did not include persons without CAA and therefore could only calculate the sensitivity of the criteria. It is likely that, especially in cohorts with a low prevalence of CAA, the use of the criteria v2.0 will lead to a higher number of false positives, especially in the possible CAA category. Another limitation is the relatively small size of our study population due to the rarity of the disease. Also, our patients were either presymptomatic or had a history of ICH. One of the aims of the criteria v2.0 was to develop criteria that are also applicable to patients with CAA with TFNEs or cognitive symptoms only. In D-CAA, cognitive symptoms or TFNEs are rare in presymptomatic mutation carriers without ICH.^3^ Future research is needed to validate the Boston criteria v2.0 in non-ICH cohorts, such as the general population and patients from transient ischemic attack (TIA) services and memory clinics. However, obtaining a reference standard in these populations may be challenging. The strengths of our study are the use of high-quality imaging and the unique patient population with a certain diagnosis of CAA.

In conclusion, our results demonstrate that the Boston criteria v2.0 improve the detection of the early asymptomatic phase of CAA compared to the previously used Boston criteria v1.5.
